# Supplementary material for: Accuracy prompts are a replicable and generalizable approach for reducing the spread of misinformation
Source: Nat Commun. 2022 Apr 28;13:2333. doi: 10.1038/s41467-022-30073-5 (PMC9051116; doi:10.1038/s41467-022-30073-5)
Supplement: Supplementary file 1 — Supplementary Information [file 41467_2022_30073_MOESM1_ESM.pdf]

Supplementary Information

Section 1. Means by condition and headline veracity.

Supplementary Table 1. Means by condition and headline veracity. Mean sharing intentions by condition and headline veracity for each experiment, and from random-effects meta-analysis.

	Control		Treatment		Just Evaluation		Platform	Content Type
	False	True	False	True	False	True		
A	0.229	0.286	0.2	0.273	0.2	0.273	MTurk	Politics
B	0.234	0.273	0.205	0.291	0.205	0.291	MTurk	Politics
C	0.263	0.301	0.217	0.319	0.217	0.319	MTurk	Politics
D	0.303	0.329	0.239	0.355			MTurk	Politics
E	0.28	0.301	0.198	0.333			MTurk	Politics
F	0.375	0.393	0.373	0.403			Lucid	Politics
G	0.368	0.383	0.338	0.391	0.318	0.38	Lucid	Politics
H	0.341	0.451	0.316	0.447	0.316	0.447	MTurk	Politics
I	0.469	0.488	0.47	0.524	0.47	0.524	Lucid	COVID-19
J	0.252	0.363	0.264	0.402	0.264	0.402	MTurk	Politics & COVID-19
K	0.41	0.448	0.391	0.448	0.391	0.448	Lucid	Politics
L	0.44	0.506	0.387	0.506	0.426	0.537	Lucid	COVID-19
M	0.392	0.46	0.373	0.453	0.362	0.451	Lucid	COVID-19
N	0.387	0.454	0.361	0.455			Lucid	COVID-19
O	0.403	0.453	0.365	0.457			Lucid	COVID-19
P	0.374	0.448	0.338	0.432	0.337	0.448	Lucid	COVID-19
Q	0.29	0.348	0.245	0.355	0.275	0.368	YouGov	COVID-19
R	0.223	0.305	0.18	0.298	0.202	0.306	YouGov	Politics
S	0.367	0.422	0.362	0.494	0.346	0.51	Lucid	COVID-19
T	0.459	0.544	0.396	0.489			Lucid	COVID-19
Meta-analytic estimate	0.341	0.396	0.309	0.404	0.307	0.405		

Section 2. Distribution of individual-level variables across subject pools.

Supplementary Table 2. Demographic variable binaries. Breakdown of % representation in various demographic binaries across both sample pools.

	Lucid/YouGov	MTurk
Female	54.4%	55.5%
White	76.0%	78.6%
Prefer Republican party over Democratic party	45.4%	35.7%
Identify with Republican Party (excludes Independents and unaffiliated voters)	45.5%	35.2%
Voted for Donald Trump in 2016	35.8%	25.9%

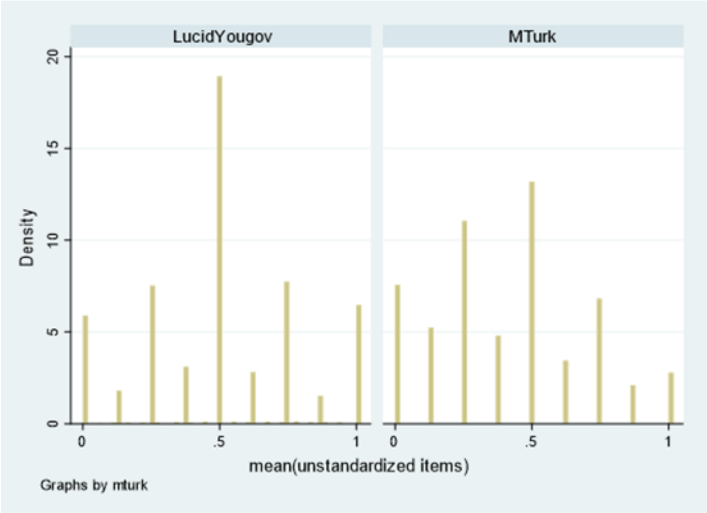

Supplementary Figure 1. Political ideology distribution. This displays the distribution of political ideology (0=maximally liberal, 1=maximally conservative) by sample pool.

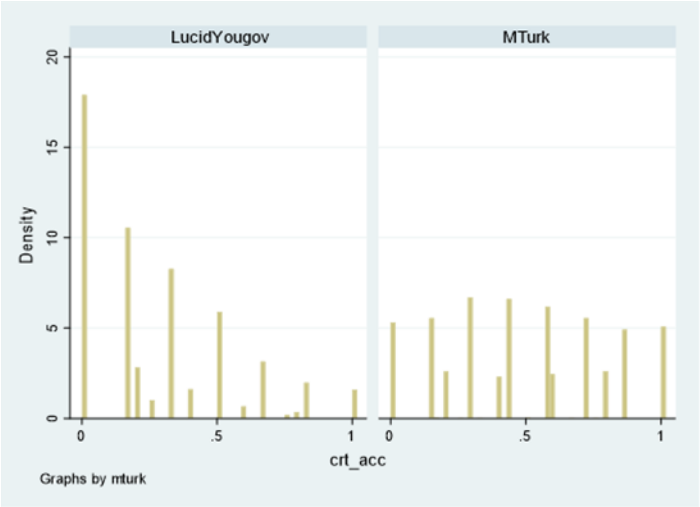

Supplementary Figure 2. Cognitive Reflection Test distribution. This displays the distribution of Cognitive Reflection Test scores (fraction of correct answers) by sample pool.

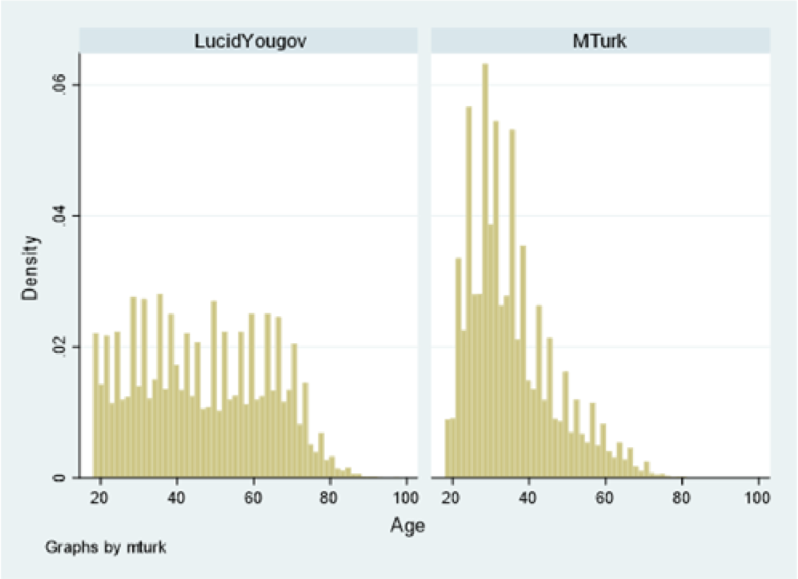

Supplementary Figure 3. Age distribution. This displays the distribution age by sample pool.

Section 3. Re-analysis of ideology in the Twitter field experiment

Here we present an analysis of the role of ideology in the accuracy prompt Twitter field experiment of [11]. There was a total of N=5,379 users in the experiment, and their ideology was estimated based on the accounts they followed using the algorithm of Barbera et al.[25]. As shown in Figure S4, the users were overwhelmingly conservative

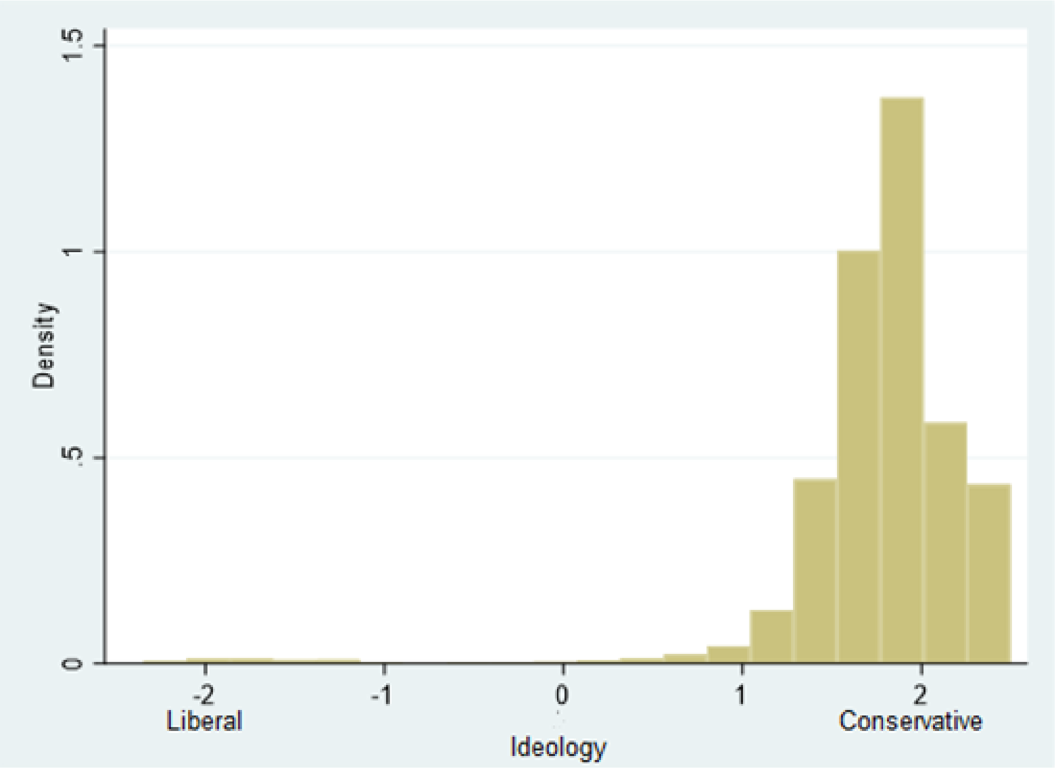

Supplementary Figure 4. Distribution of ideology scores for Twitter users in the field experiment. Ideology was estimated based on the accounts they followed using the algorithm of Barbera et al.

Given the extreme left skew of the distribution of ideology scores, we follow the same approach used in [11] for handling extreme values and winsorize ideology at the lower 95th percentile. We then look at the interaction between a "post-treatment" dummy and ideology when predicting the quality of news links shared by the users. We follow the main text models of [11] and analyze retweets without comment, include links to both opinion and non-opinion articles, and exclude data from the day on which a technical issue led to a randomization failure. We focus on the most straight-forward outcome measure, the average relative quality of links retweeted in a given user-day, and the model specification reported in the main text which includes wave fixed effects and calculates P values using Fisherian Randomization Inference (comparing t-statistics across 500 permutations). This model finds no significant interaction between ideology and the post-

treatment dummy (p_{FRI} = 0.97); the model is visualized in Figure S5. As shown in Table S2, we also find no significant interaction when using other model specifications, or the outcome measure of summed average quality of links retweeted.

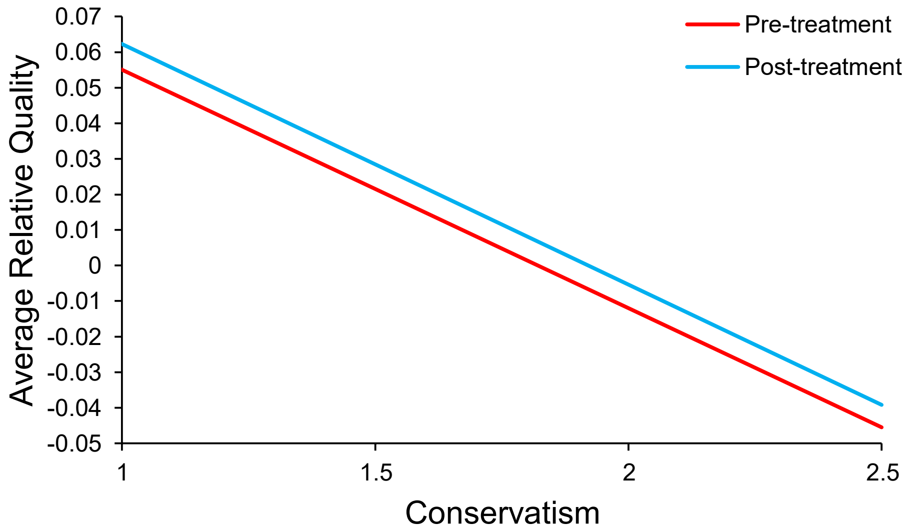

Supplementary Figure 5. Average relative quality as a function of experimental condition across the spectrum of political ideology. Predicted average relative quality score based on ideology and pre- versus post-treatment in the Twitter field experiment.

Supplementary Table 3. Coefficients and two-sided p-values for the interaction between ideology and the post-treatment dummy. This was computed for various model specifications and outcome variables, derived from the regression models described in the text. No adjustments were made for multiple comparisons.

**Model Specification**	**Average relative tweet quality**			**Summed relative tweet quality**		
	**b**	**Regression p**	**FRI p**	**b**	**Regression p**	**FRI p**
Wave Fixed Effects	-0.001	0.95	0.97	-0.013	0.39	0.76
Wave Post-Stratification	-0.001	0.95	0.97	-0.013	0.38	0.76
Date Fixed Effects	-0.001	0.93	0.96	-0.013	0.37	0.76
Date Post-Stratification	-0.001	0.96	0.97	-0.013	0.39	0.75

Section 4. Moderation of accuracy prompt effect on discernment by ideology and partisanship considering only the Evaluation treatment.

Supplementary Table 4. Coefficients, *z* values, and two-sided *p* values for the individual-level difference moderation analyses. These were derived from the regression models described in the text, using only the Evaluation treatment. Shown are the coefficients for the 3-way interaction between headline veracity, condition, and the individual difference - which captures the extent to which the individual difference moderates the treatment effect on sharing discernment. Results are shown separately for the more representative samples from Lucid or YouGov, versus the convenience samples from MTurk. Coefficients with $p<0.05$ are bolded. No adjustments were made for multiple comparisons.

Evaluation Treatment Only	Moderation of accuracy prompt effect on discernment					
	Lucid/YouGov			MTurk		
	b	*z*	*p*	*b*	*z*	*p*
Female	-0.011	-0.931	0.352	-0.009	-0.639	0.523
White	0.002	0.198	0.843	-0.020	-1.186	0.236
Age	**0.001**	**2.099**	**0.036**	-0.000	-0.096	0.924
College degree	-0.016	-1.146	0.252	-0.001	-0.047	0.963
Conservatism	-0.006	-0.277	0.782	**-0.045**	**-1.992**	**0.046**
Republican	-0.012	-1.201	0.230	**-0.020**	**-2.584**	**0.010**
Republican (No Independents)	-0.015	-1.355	0.175	**-0.025**	**-2.645**	**0.008**
Trump 2016 Voter	-0.006	-0.416	0.677	**-0.024**	**-2.097**	**0.036**
Value Accuracy	0.009	0.358	0.720	**0.036**	**3.125**	**0.002**
Cognitive Reflection (CRT)	0.028	1.020	0.308	**0.033**	**2.432**	**0.015**
Attentiveness	**0.048**	**2.459**	**0.014**			

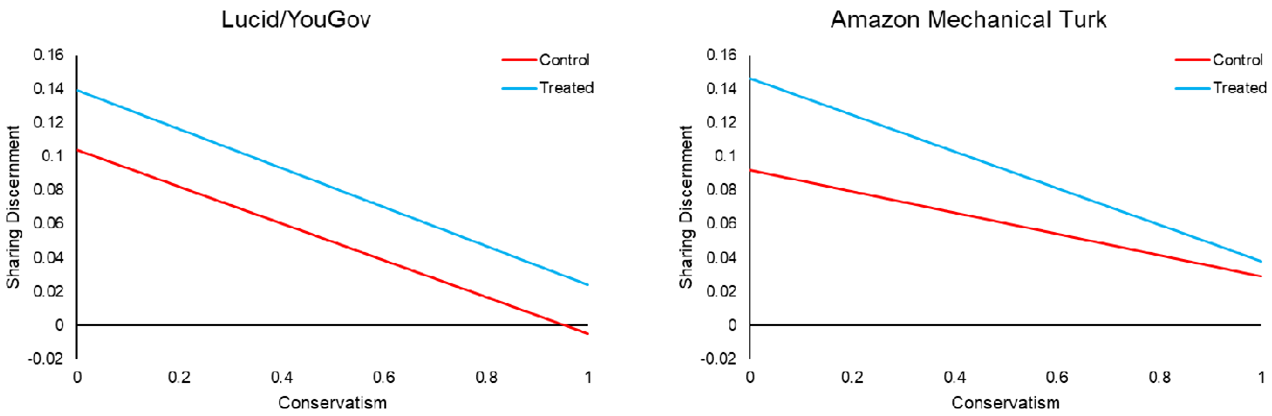

Supplementary Figure 5. Effect of the Evaluation treatment on sharing discernment as a function of liberal versus conservative ideology. The model fits for discernment in control and treatment, based on meta-analytic estimates of model coefficients, are shown with solid lines.

More representative samples from Lucid and YouGov are shown in the left panel; convenience samples from Amazon Mechanical Turk are shown in the right panel.

Section 5. Meta-regression for individual differences

Supplementary Table 5. Coefficients and p values from meta-regressions predicting individual-level difference moderation using platform, news type, and baseline discernment. The left half of the table shows the coefficients when predicting the coefficient for the 3-way interaction between headline veracity, condition, and the individual difference - which captures the extent to which the individual difference moderates the treatment effect on sharing discernment. The right half of the table shows the coefficients when predicting the coefficient on the 2-way interaction between headline veracity and the individual difference - which captures how the individual difference relates to baseline sharing discernment in the control condition.

	Moderation of accuracy prompt effect on discernment						Relationship with baseline discernment in control					
	Platform (1=Mturk)		Type (1=Politics)		Baseline discernment		Platform (1=Mturk)		Type (1=Politics)		Baseline discernment	
	b	p	b	p	b	p	b	p	b	p	b	p
Female	3.6E-03	0.851	-6.1E-03	0.748	-5.5E-02	0.842	-1.4E-02	0.375	-8.9E-03	0.586	1.6E-01	0.478
White	-7.0E-03	0.769	-1.6E-02	0.386	-2.7E-01	0.348	2.1E-02	0.329	2.6E-02	0.114	1.6E-01	0.513
Age	6.3E-05	0.9	-6.8E-04	0.145	-9.6E-03	0.209	-3.5E-04	0.582	-3.9E-04	0.523	1.4E-02	0.146
College degree	2.0E-04	0.992	-2.4E-02	0.254	3.4E-01	0.259	-7.7E-03	0.686	3.0E-03	0.873	-2.9E-01	0.297
Conservatism	-5.4E-02	0.123	1.9E-02	0.581	4.0E-01	0.399	-8.0E-03	0.893	1.0E-01	0.069	-5.3E-01	0.509
Republican	-2.2E-02	0.078	1.0E-02	0.37	1.4E-01	0.413	1.0E-02	0.748	7.0E-02	0.025	-5.9E-01	0.168
Republican (No Independents)	-1.8E-02	0.198	-2.5E-04	0.985	2.4E-01	0.223	1.7E-02	0.615	8.5E-02	0.013	-9.0E-01	0.068
Trump 2016 Voter	-4.3E-02	0.033	2.8E-02	0.141	4.4E-02	0.866	2.5E-02	0.418	5.4E-02	0.064	-8.6E-01	0.056
Value Accuracy	2.0E-02	0.411	-1.8E-03	0.946	5.2E-02	0.897	2.4E-02	0.385	-4.9E-02	0.128	-3.7E-01	0.415
Cognitive Reflection (CRT)	8.0E-04	0.984	-2.4E-02	0.551	3.8E-01	0.43	-2.9E-02	0.271	3.9E-02	0.186	5.4E-01	0.198

Section 6. Including research from other groups

Here we consider the robustness of our main meta-analytic results to including accuracy prompt studies conducted by other groups that meet our inclusion criteria. Doing so yields only one additional eligible study, Roozenbeek et al. (2021). The accuracy prompt experiment of Byles et al. (2021) is ineligible because it was conducted in 2021; the accuracy prompt experiment of Pretus et al. (2021) is ineligible because it was conducted outside of the U.S., and the literacy tips experiment of Guess et al. (2020) is ineligible because the survey asked about accuracy on every item. Here we reproduce our main analyses including Roozenbeek et al. (2021) (labeled study X, using the full "pooled" dataset from both rounds of data collection in that paper); the results are virtually identical to the analyses in the main text.

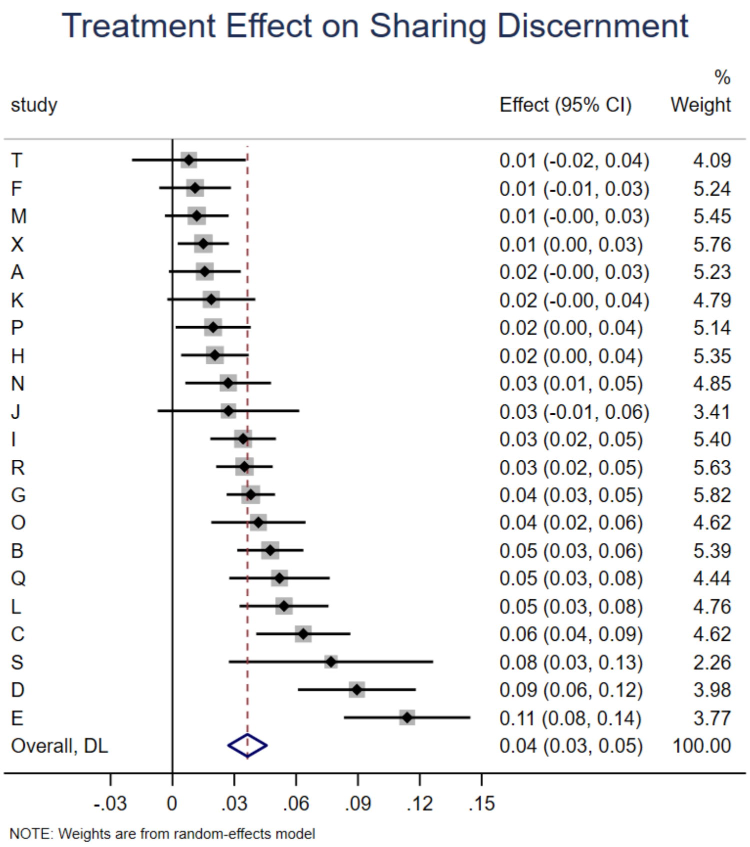

Supplementary Figure 6. Meta-analytic estimate (via random effects meta-analysis) of the effect of accuracy prompts on sharing discernment across the 20 experiments analyzed in this paper plus Roozenbeek et al. (2021). The coefficient on the interaction between condition and headline veracity and 95% confidence interval are shown for each study, and the meta-analytic estimate is shown with the red dotted line and blue diamond (positive values indicate that the treatment increased sharing discernment).

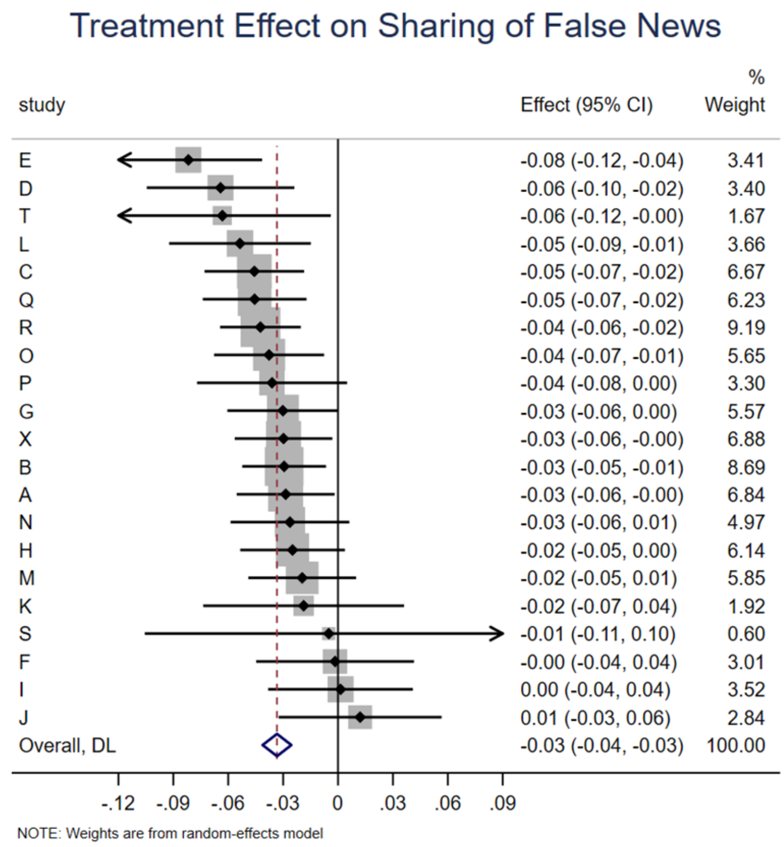

Supplementary Figure 7. Meta-analytic estimate (via random effects meta-analysis) of the effect of accuracy prompts on sharing of false news across the 20 experiments analyzed in this paper plus Roozenbeek et al. (2021). The coefficient on the condition dummy (which captures the effect of the treatment on sharing of false headlines) and 95% confidence interval are shown for each study, and the meta-analytic estimate is shown with the red dotted line and blue diamond.

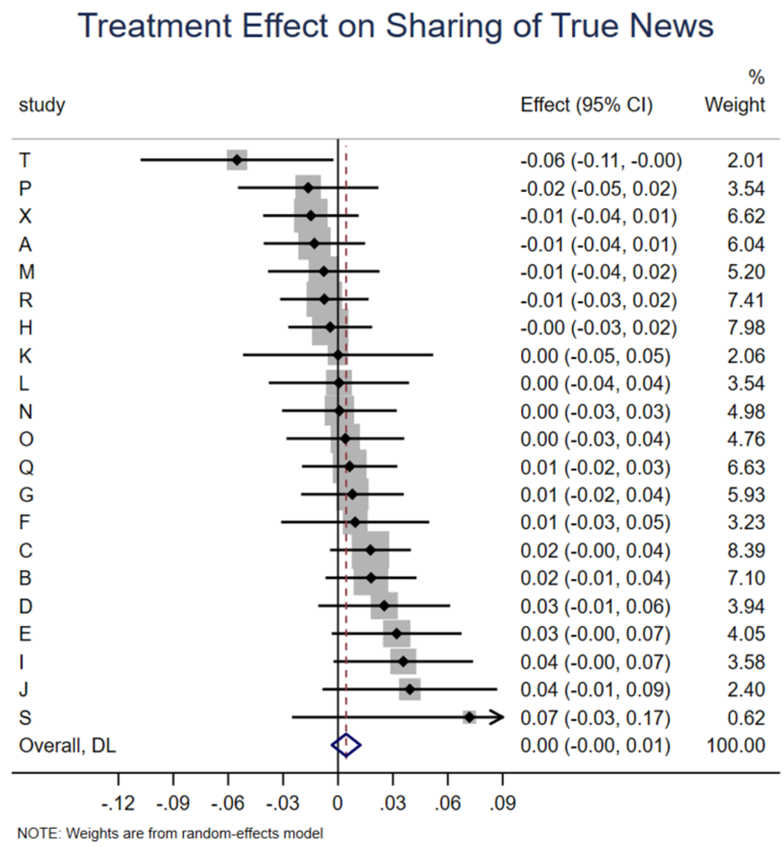

Supplementary Figure 8. Meta-analytic estimate (via random effects meta-analysis) of the effect of accuracy prompts on sharing of true news across the 20 experiments analyzed in this paper plus Roozenbeek et al. (2021). The coefficient on the condition dummy when analyzing true headlines and 95% confidence interval are shown for each study, and the meta-analytic estimate is shown with the red dotted line and blue diamond.
