# Supplementary material for: Accuracy prompts are a replicable and generalizable approach for reducing the spread of misinformation
Source: Nat Commun. 2022 Apr 28;13:2333. doi: 10.1038/s41467-022-30073-5 (PMC9051116; doi:10.1038/s41467-022-30073-5)
Supplement: Supplementary file 3 — Reporting Summary [file 41467_2022_30073_MOESM3_ESM.pdf]

nature portfolio

Corresponding author(s): Gordon Pennycook, David Rand

Last updated by author(s): Mar 5, 2022

Reporting Summary

Nature Portfolio wishes to improve the reproducibility of the work that we publish. This form provides structure for consistency and transparency in reporting. For further information on Nature Portfolio policies, see our Editorial Policies and the Editorial Policy Checklist.

Statistics

For all statistical analyses, confirm that the following items are present in the figure legend, table legend, main text, or Methods section.

n/a	Confirmed	
☐	☒	The exact sample size (*n*) for each experimental group/condition, given as a discrete number and unit of measurement
☐	☒	A statement on whether measurements were taken from distinct samples or whether the same sample was measured repeatedly
☐	☒	The statistical test(s) used AND whether they are one- or two-sided<br>*Only common tests should be described solely by name; describe more complex techniques in the Methods section.*
☒	☐	A description of all covariates tested
☐	☒	A description of any assumptions or corrections, such as tests of normality and adjustment for multiple comparisons
☐	☒	A full description of the statistical parameters including central tendency (e.g. means) or other basic estimates (e.g. regression coefficient) AND variation (e.g. standard deviation) or associated estimates of uncertainty (e.g. confidence intervals)
☐	☒	For null hypothesis testing, the test statistic (e.g. *F*, *t*, *r*) with confidence intervals, effect sizes, degrees of freedom and *P* value noted<br>*Give P values as exact values whenever suitable.*
☒	☐	For Bayesian analysis, information on the choice of priors and Markov chain Monte Carlo settings
☒	☐	For hierarchical and complex designs, identification of the appropriate level for tests and full reporting of outcomes
☐	☒	Estimates of effect sizes (e.g. Cohen's *d*, Pearson's *r*), indicating how they were calculated

Our web collection on statistics for biologists contains articles on many of the points above.

Software and code

Policy information about availability of computer code

Data collection	This is a meta-analysis, data was collected from 20 previous experiments
Data analysis	Stata was used

For manuscripts utilizing custom algorithms or software that are central to the research but not yet described in published literature, software must be made available to editors and reviewers. We strongly encourage code deposition in a community repository (e.g. GitHub). See the Nature Portfolio guidelines for submitting code & software for further information.

Data

Policy information about availability of data

All manuscripts must include a data availability statement. This statement should provide the following information, where applicable:

- Accession codes, unique identifiers, or web links for publicly available datasets
- A description of any restrictions on data availability
- For clinical datasets or third party data, please ensure that the statement adheres to our policy

Data and code is available here: https://osf.io/4mv9z/

Field-specific reporting

Please select the one below that is the best fit for your research. If you are not sure, read the appropriate sections before making your selection.

☐ Life sciences ☒ Behavioural & social sciences ☐ Ecological, evolutionary & environmental sciences

For a reference copy of the document with all sections, see nature.com/documents/nr-reporting-summary-flat.pdf

Behavioural & social sciences study design

All studies must disclose on these points even when the disclosure is negative.

Study description	Meta-analysis of quantitative survey experiments
Research sample	Online panels of participants via Mechanical Turk, Lucid, and YouGov. Some samples are convenience samples and others used quota-matching for national representativeness. Samples were selected based off of the tradeoffs between cost, representativeness, and attentiveness.
Sampling strategy	We report all accuracy prompt experiments completed by our lab until the end of 2020. The individual studies obtained participants using online survey platforms where participants sign up for studies in exchange for compensation. Convenience samples were open to any social media user whereas the quota-matched representative studies attempted to match U.S. demographics. Sample sizes were chosen based on the maximum amount of money that we wished to spend on each study while maintaining high statistical power. The individual studies included in the meta-analysis vary in their sample sizes, but almost all are highly powered. The meta-analysis itself is very highly powered as it includes >20,000 participants.
Data collection	Original data was collected in online survey experiments using Qualtrics survey software. The studies were completed online, so there was no interaction between researcher and participant (beyond the text in the study).
Timing	Studies were conducted between 2017 and 2020.
Data exclusions	No exclusions
Non-participation	This varies from study to study, but the average dropout rate across studies is 7.2%.
Randomization	Participants were randomly assigned to conditions for each study.

Reporting for specific materials, systems and methods

We require information from authors about some types of materials, experimental systems and methods used in many studies. Here, indicate whether each material, system or method listed is relevant to your study. If you are not sure if a list item applies to your research, read the appropriate section before selecting a response.

Materials & experimental systems

n/a	Involved in the study	
☒	☐	Antibodies
☒	☐	Eukaryotic cell lines
☒	☐	Palaeontology and archaeology
☒	☐	Animals and other organisms
☐	☒	Human research participants
☒	☐	Clinical data
☒	☐	Dual use research of concern

Methods

n/a	Involved in the study	
☒	☐	ChIP-seq
☒	☐	Flow cytometry
☒	☐	MRI-based neuroimaging

Human research participants

Policy information about studies involving human research participants

Population characteristics	Varies from study to study.
Recruitment	Each study recruiting participants using online survey panels. Participants volunteered for our studies in exchange for financial compensation. This self-selection may impact our results in ways that are difficult to estimate (knowing the impact of this self-selection requires having participants who do not wish to complete studies decide to complete studies, which they don't wish to do).
Ethics oversight	This is a meta-analysis, but all individual studies had ethics approval (Yale University, MIT, or University of Regina)

March 2021

Note that full information on the approval of the study protocol must also be provided in the manuscript.
